# Risk for primary cephalosporin resistance in Gram-negative bacteremia

**DOI:** 10.1017/ash.2023.202

**Published:** 2023-07-10

**Authors:** Princy John, Sona Shahbazian, William D. Lainhart, Justin Hayes, Brian Mochon, David E. Nix

**Affiliations:** 1 Banner Health; 2 College of Medicine, University of Arizona, Tucson, Arizona; 3 Department of Pathology & Laboratory Medicine, University of Arizona, Tucson, Arizona; 4 Department of Pharmacy Practice & Science, University of Arizona, Tucson, Arizona

## Abstract

**Objective:**

This study aimed to examine the clinical risk factors for cephalosporin resistance in patients with Gram-negative bacteremia caused by *Escherichia coli* (EC), *Klebsiella pneumoniae* (KP), *Enterobacter cloacae* (ENC), and *Pseudomonas aeruginosa* (PS).

**Methods:**

This retrospective cohort study included 400 adults with Gram-negative bacteremia. The goal was to review 100 cases involving each species and approximately half resistant and half susceptible to first-line cephalosporins, ceftriaxone (EC or KP), or cefepime (ENC or PS). Logistic regression was used to identify factors predictive of resistance.

**Results:**

A total of 378 cases of Gram-negative bacteremia were included in the analysis. Multivariate analysis identified significant risk factors for resistance, including admission from a chronic care hospital, skilled nursing facility, or having a history of infection within the prior 6 months (OR 3.00, *P* < .0001), requirement for mechanical ventilation (OR 3.76, *P* < .0001), presence of hemiplegia (OR 3.54, *P* = .0304), and presence of a connective tissue disease (OR 3.77, *P* = .0291).

**Conclusions:**

Patients without the identified risk factors should be strongly considered for receiving ceftriaxone or cefepime rather than carbapenems and newer broad-spectrum agents.

## Introduction

In 2019, the Centers for Disease Control and Prevention released a report of antibiotic resistance threats in the United States stating that more than 2.8 million infections and over 35,000 deaths annually are due to antimicrobial-resistant pathogens.^1^ A comprehensive global analysis from over 200 countries and territories concluded that bacterial resistance was responsible for an estimated 4.95 million deaths, with 1.27 million deaths directly attributable to bacterial resistance.^2^ Moreover, *Clostridioides difficile* infections are related to antibiotic use, resulting in 223,900 illnesses and 12,800 deaths annually.^3^ Successful treatment of an infection depends on timely administration of effective antibiotics. At the same time, the overuse of antibiotics contributes to antimicrobial resistance (AMR) and creates pressure to use agents with a broader antimicrobial spectrum and increased potency. Broader-spectrum agents promote the evolution of new mechanisms to overcome the actions of antimicrobials. Without behavioral change in how antibiotics are prescribed and used, AMR will remain a major threat, ultimately compromising our ability to treat common bacterial infections.^3^ The COVID-19 pandemic served as an external force to increase antibiotic use with a resulting 15% increase in antimicrobial-resistant infections in hospitals.^4^

Several investigators have attempted to identify risk factors for AMR in specific clinical settings. A literature review of five studies showed that risk factors contributing to pneumonia caused by a multidrug-resistant organism (MDRO) included previous hospital admission of 2 or more days within the past 90 days, admission from a skilled nursing facility, and antibiotic therapy within the last 90 days.^5^ Patients with Gram-negative bloodstream infections (BSI) with fluoroquinolone-nonsusceptible bacilli were studied in a retrospective study. Multivariate logistic regression identified independent risk factors for fluoroquinolone resistance (FQ-R) including male sex, diabetes mellitus, residence at a skilled nursing facility, undergoing an outpatient procedure within the previous 30 days, or fluoroquinolone use within 180 days prior to admission.^6^ Another retrospective study at a major academic hospital system determined risk factors for carbapenem-resistant Gram-negative rods. Risk factors for antipseudomonal carbapenem resistance (CR) included male sex, admission from another healthcare facility, receipt of mechanical ventilation, receipt of any carbapenem in the previous 30 days, and receipt of any anti-MRSA agent in the prior 30 days.^7^ The last two studies both included a scoring rule.^6,7^ Clinical risk scores were developed to estimate the risk of CR or extensive β-lactam resistance (EBR) among hospitalized adult patients with *Pseudomonas aeruginosa* infections. For CR, points were assigned for the following criteria: transfer from a skilled nursing facility, tracheostomy, prior *P. aeruginosa* infection within 30 days, previous hospitalization within 6 months, or receipt of antibiotics within 30 days. Similar risk factors and scoring have been reported for the EBR *P. aeruginosa*.^8^

In this study, we focused on *P. aeruginosa* (PS), *Escherichia coli* (EC), *Klebsiella pneumoniae* (KP), and *Enterobacter cloacae* (ENC) because they are common Gram-negative organisms associated with bloodstream infections and encompass a variety of resistance mechanisms. Ceftriaxone is considered the first-line antimicrobial agent for the treatment of EC or KP infection, and cefepime is considered the first-line treatment for ENC or PS. Extended-spectrum cephalosporins or piperacillin-tazobactam are usually appropriate for the treatment of these infections. However, due to increasing ESBL production in Enterobacterales, many providers prescribe carbapenems, leading to their overuse. Implementing limits on carbapenem use is a major goal in antimicrobial stewardship. Therefore, the goal of this study was to determine risk factors for resistance to these first-line antimicrobial drugs, and in the absence of “risk factors,” justification to avoid carbapenem use.

## Methods

### Setting and population

The study was conducted as a multicenter, case–control study performed across all Banner Health hospitals (27 facilities in six Western states). The Banner Health Institutional Review Board approved this study and waived the requirement for informed consent. All patients were ≥ 18 years of age and were hospitalized between July 01, 2016 and June 30, 2021, with bacteremia involving EC, KP, ENC, or PS. Patients with an artificial heart, a left ventricular assistive device, or a similar implanted device with an external component were excluded. The microbiology laboratory provided a list of all positive blood cultures for the four species listed above. Starting with the most recent year and then moving backward, we attempted to identify 50 resistant and 50 susceptible strains for each species. If more than 50 species were found, 50 were randomly selected from the available pool. Random samples were obtained using Proc Survey Select available in SAS (SAS Institute, Cary, NC, USA). Primary resistance was defined as resistance to ceftriaxone for EC and KP and resistance to cefepime for ENC and PS. Susceptibility was determined using the CLSI guidelines.^9^

### Data collection and analysis

Pertinent demographics, history of prior infection, culture and susceptibility data, prior hospitalizations, and comorbidities were obtained from the electronic medical records. For prior history, we examined multiple sources, including admission/discharge physician notes, Emergency Department notes, and clinic notes. The Charlson comorbidity index was used to characterize the health of the population.^10^ We used definitions from the Charlson Comorbidity Index that were used to define and categorize disease states, such as chronic renal disease, diabetes, and liver disease. The presence or absence of variables was determined within 24 hours prior to the index blood culture. The study data were collected and managed using REDcap electronic data capture tools hosted at the University of Arizona.^11,12^

All statistical evaluations were performed using SAS software. The patient had a bloodstream infection with a target organism resistant to ceftriaxone or cefepime, as previously stated, while the control patients had a target organism susceptible to the cephalosporin. Interval data were compared using the t-test or Wilcoxon signed-rank test (if not normally distributed). The χ^2^ test was used for ordinal data. To evaluate potential risk factors, we used logistic regression implemented using Proc Logistic in SAS.

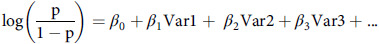




The logistic regression model building included stepwise forward and backward elimination runs to ensure that only the important risk factors were captured. Pairwise correlations were examined for candidate factors, and biological rationale was used to select between two correlated (*r* ^2^ > 0.5) parameters. Model discrimination was performed using the receiver operating characteristic area under the curve and Akaike’s Information Criteria. Concordance is calculated as (number of concordant pairs + number of ties)/total number × 100%.

The system antibiogram specific for blood cultures and the susceptibility of the four organisms was obtained from laboratory records, providing the total number of isolates and % resistance for the system. Classification of intermediate versus susceptible dose-dependent changed during the study; therefore, we included only S or R determinations for ENC. For each of the four species, there were a total number of susceptible isolates and a total number of “resistant” isolates over the study period. The model dataset included 100 or fewer patients for each species classified as either susceptible (S) or resistant (R) along with independent variables and predicted probabilities of R. If, for example, based on the system antibiogram, there were a total of 750 isolates with EC susceptible to ceftriaxone and 80 with EC resistant to ceftriaxone, the 50 cases in the study dataset will be replicated 15 times to make 750 records. Thirty cases were randomly selected from the resistant study dataset and added to the original 50 cases to obtain 80 cases. Susceptible and resistant cases were combined (750+80) resulting in 830 cases with a 9.6% observed resistance rate. The model-predicted probabilities were summarized to explore how the model could discriminate between what the numbers would look like at the system level.

## Results

A total of 406 cases were identified during screening. Fourteen were excluded due to age < 18 years, seven patients were excluded as extra EC-S cases, and seven cases were excluded as intermediate to cefepime before the categorization was changed to susceptible dose-dependent (SDD). For PS, 10 strains were classified as intermediate to cefepime, and these were grouped with resistant isolates. After exclusion, 378 patients were eligible for the study. Fifty susceptible cases were obtained for each organism, with the exception of KP-S (*n* = 51). Of the resistant isolates, only 27 EC-R isolates were available after the removal of the excluded patients. The baseline characteristics of the patients in the two study groups are presented in Table 1. The predicted 10-year survival based on the Charlson Comorbidity index was similar, at approximately 50% for both groups.

**Table 1.** Demographic summary

Variable	Resistant group		Susceptible group		*P* value
	n	Mean ± SD	n	Mean ± SD	
Age	177	58.4 ± 17.1	201	59.3 ± 17.1	.607
Weight	177	81.1 ± 24.5	201	82.9 ± 29.7	.530
BMI	…	28.4 ± 8.75	…	28.7 ± 8.86	.761
CCI	…	4.51 ± 3.05	…	4.25 ± 2.92	.122
Sex (% Female)	…	48.6%	…	45.8%	.607
Baseline sCr	177	0.89 ± 0.49	201	0.90 ± 0.38	.887
30-day mortality^a^		47/155		44/169	.446

Note. BMI, body mass index; CCI, Charlson comorbidity index; sCr, serum creatinine.

a 30-day mortality unknown for 22 and 32 cases, respectively.

The characteristics and comorbidities of the patients found to be associated with resistance (χ^2^ of Fisher’s exact test, *P* < .15) are shown in Table 2. Fifteen variables were associated with primary resistance based on the univariate analysis. Residence in a skilled nursing facility or chronic care hospital prior to admission was associated with an increased risk compared to those living at home or in an assisted living arrangement. With regard to vascular access, the presence of a peripherally inserted central catheter or tunneled central catheter was responsible for the increased risk compared to other access modalities. Data on hospital admissions prior to the index admission were collected at various time points (0–30 days, 31–60 days, 61–90 days, and 91–180 days). Since there was no difference between these subgroups, any hospital admission within the past six months was evaluated. A history of infections within the previous 6 months was more strongly associated with resistance, and this association was notable for a history of urinary tract infection, pneumonia, skin and skin structure infection, or bacteremia. Immunosuppression as a whole was not associated with resistance, and the only individual type of immunosuppression associated with resistance was cancer chemotherapy.

**Table 2.** Odds ratio values for individual factors (univariate) with *P* < .15

Variable	OR	B	*P* value
Chronic kidney disease^a^	1.837	0.3041	.0095
SNF/CCH	4.565	0.7592	< .0001
Home health care	2.234	0.4018	.0745
Infection (6 months)	3.027	0.5538	< .0001
Hospitalization (6 months)	1.392	0.1655	.122
Peripheral vascular disease	2.084	0.3672	.0095
Cerebral vascular accident	2.200	0.3943	.0435
Connective tissue disease	4.230	0.7211	.0124
Liver disease	1.550	0.2191	.1002
Hemiparesis	5.922	0.8893	.0015
COVID-19	2.267	0.2129	.0547
Mechanical ventilation	3.667	0.6497	< .0001
Central catheter	2.674	0.4917	.0001
Organism (ENC vs EC)	0.540	–0.4572	.0215
Charlson comorbidity index (> 5)	1.454	0.1873	.0706

Note. CCH, chronic care hospital; SNF, skilled nursing facility; ENC, *Enterobacter cloacae* complex; EC, *Escherichia coli*.

a Chronic kidney disease defined as eGFR < 60 ml/min/1.73 m2, (6 months in parenthesis refers to period 6 months prior to admission).

Table 3 presents the final model for predicting primary resistance. A strong relative risk was detected for the presence of infection within the past 6 months or transfer from a skilled nursing facility or chronic care hospital. The latter two were combined (SNF or CCH) because they are exclusive to one another and impart a similar risk of resistance. Next, both infection within the previous 6 months and transfer from an SNF or CCH were strongly associated with the risk of resistance individually, but not independently, in the multivariate model. These variables were highly correlated (*r* = 0.99). The model was slightly improved by considering infection within 6 months or admission from an SNF or CCH as a single predictor variable. The final model was based on information about Gram-negative bacteremia without knowledge of the species. Then, the species was added as an additional factor, and the p-value was 0.0055. The pairwise OR and p values were KP vs. EC (OR=0.545, *P* = .793), ENC vs. EC (OR=0.289, *P* = .0106), and PS vs. EC (OR=0.456, *P* = .536). ENC was the least likely species to be detected as resistant in patients without concomitant risk factors, and this appeared to account for the lower OR.

**Table 3.** Final model

Variable	OR	β	*P* value
Intercept	…	1.0528	< .0001
Mechanical ventilator	3.762	0.6624	< .0001
SNF or CCH or infection	2.995	1.097	< .0001
Connective tissue disease	3.774	0.6641	.0291
Hemiplegia	3.539	0.6320	.0304

Note. SNF, skilled nursing facility; CCH, chronic care hospital; Infection, infection history in the last six months prior to index infection.

ROC area = 0.7056.

Concordance = 84.3% (concordant pairs + ties).

Based on the system antibiogram (adults and blood culture-specific), primary resistance (total N) was 15.2% (10,276) for EC, 12.2% (3,546) for KP, 9.5% (792) for ENC, and 8.9% (1,365) for PS. Extrapolation from our study population predicted the level of resistance in patients without any risk factors compared with patients with at least one risk factor. For EC, the resistance was expected to be 13.8% compared to 32.0%. For KP, the expected resistance was 5.9% versus 54%, respectively. For Enc and Ps, resistance is expected in 5.8%–6.3% of patients without any risk factor and 22%–24% of patients with at least one risk factor. Table 4 lists the breakdown of the model-predicted probability of resistance and observed resistance by risk stratification.

**Table 4.** Model probability based on risk factors present and expected % resistance extrapolated to actual numbers of blood isolates

Risk factor						AST result
Vent	Fac-Infect	CTD	Hemiplegia	n	Probability of resistance	% Resistant
0	0	0	0	182	0.288	29.7
1	0	0	0	36	0.603	61.1
0	1	0	0	101	0.548	51.5
0	0	1	0	6	0.604	…
0	0	0	1	2	0.589	…
1	1	0	0	20	0.820	90.0
1	0	1	0	3	0.852	…
1	0	0	1	2	0.843	…
0	1	1	0	3	0.820	…
0	1	0	1	13	0.811	92.3
0	0	1	1	1	0.844	…
1	1	1	0	4	0.945	…
1	1	0	1	2	0.942	…
1	1	1	1	1	0.984	…

Note. Vent, mechanically ventilated; Fac-Infect, arriving from a skilled nursing facility or chronic care hospital or a history of infection within the previous 6 months; CTD, connective tissue disease.

The percent resistance was only presented for cases with n > 10. Note that this is the modeled dataset, where the overall resistance was 46.8%.

## Discussion

Four risk factors were identified in our study that increased the odds of encountering primary resistance to ceftriaxone (EC or KP) or cefepime (ENC or PS). These factors included mechanical ventilation, admission from SNF/CCH or infection within the last 6 months, history of connective tissue disease, and history of hemiplegia. Initially, a score was developed to characterize the risk of resistance; however, our goal was to identify the subpopulation with the lowest risk of resistance to justify the use of first-line antimicrobial agents.

A previous study was performed in 192 adults with cancer who developed Enterobacter bacteremia. The identified species included *E. cloacae* (67.7%), *Klebsiella* (formally Enterobacter) *aerogenes* (29.2%), and four other *Enterobacter* sp. Of the Enterobacter isolates, 27.6% were reported to be resistant to extended-spectrum cephalosporins (ESC) and the remainder were susceptible. The authors found that previous exposure to an ESC, tumor progression, recent surgery, and nosocomial acquisition were risk factors for ESC resistance. Lower respiratory tract infection, tumor progression, septic shock, and *E. aerogenes* bacteremia were identified as risk factors for mortality.^13^ In contrast, we assume that *E. cloacae* and *K. aerogenes* are resistant and report as such, and cefepime is an acceptable therapy if the isolate is susceptible *in vitro*. Another study included patients with community-onset *Escherichia coli* bacteremia in Taiwan. The incidence of ESC resistance was 9.6% in bacteremia isolates. Among the ESC-resistant isolates, appropriate empiric therapy was administered to 45.9% of 133 patients, compared to 92.6% of 543 patients with susceptible isolates. ESC resistance was associated with increased 14-day mortality and longer length of stay. Factors associated with ESC resistance included hospitalization within the past year, exposure to antibiotics within the past 15 days, residence in a long-term care facility, underlying genitourinary disease, and presence of an implantable intravenous port.^14^

Antimicrobial stewardship requires a balance between selecting active empiric therapy and avoiding overuse of protected antimicrobial agents. Ensuring active empiric therapy is particularly important for patients with septic shock.^15^ Many antibiotics, including ceftriaxone and cefepime, qualify as broad-spectrum antibiotics and are appropriate for empirical coverage. Among a large population of surgical patients, patients who received an appropriate initial antibiotic treatment (based on susceptibility to the pathogen(s)) did not demonstrate improved survival over patients who had a pathogen resistant to the empiric therapy.^16^ In 670 patients with Gram-negative bacteremia, inappropriate empiric treatment (pathogen resistant) was not significantly associated with 7-day or 30-day all-cause, mortality.^17^ Likewise, patients with community-onset bacteremia due to ESBL-producing *E. coli* or *K. pneumonia* did not exhibit increased mortality as long as they received a carbapenem as definitive therapy.^18^ In sepsis due to a urine source caused by *E. coli* susceptible or resistant to a third-generation cephalosporin, mortality was higher in the group with resistant isolates; however, mortality was not associated with inappropriate empiric therapy.^19^ In a systematic review and meta-analysis, inappropriate empirical antibiotic therapy was associated with increased mortality, but 42.3% of patients had septic shock. The odds ratio for mortality was 22.3 for presence of septic shock and 1.83 for inappropriate empiric therapy.^20^ Additionally, another study demonstrated that inappropriate empiric treatment contributes to worse outcomes in patients with hospital acquired pneumonia, ventilated hospital acquired pneumonia, and ventilator-associated pneumonia. The organism with the highest incidence of inappropriate empiric therapy was *P. aeruginosa* (24.2%).^21^

The goal of this study was to identify a subpopulation at low risk for primary resistance, given the more common Gram-negative organisms recovered from blood cultures. Patients with urinary tract infections and intra-abdominal infections are most likely to have Enterobacterales infections (eg, *E. coli* and *K. pneumoniae*), and ceftriaxone could be selected in the absence of risk factors (Table 4). If the patient had risk factors for pseudomonal infection or hospital-acquired Gram-negative infection, cefepime could be selected in the absence of risk factors. Owing to the high early mortality risk, we suggest not applying this recommendation to patients with septic shock. The restriction of carbapenem use has already been implemented in many institutions. Carbapenems are often restricted to use in patients with worsening infection when receiving a cephalosporin or piperacillin-tazobactam, documented resistance to empiric therapy, history of ESBL-producing Gram-negative infection, and/or approval by an infectious disease specialist or antimicrobial stewardship program.^22^ Another strategy is to require infectious disease consultation for extending carbapenem use beyond 72 hours. Interestingly, this strategy resulted in a 45% to 65% reduction in the initial use of a carbapenem.^23^

As this study was based on a retrospective review of the electronic health records, a noteworthy limitation is that of missing or inaccurate data. Specifically, prior hospitalizations may have taken place in a different hospital system, or there may be missing past medical history. This study focused on the four most common pathogens of Gram-negative bacteremia but did not capture data on patients with other Gram-negative organisms. We fell short of the planned number of cefepime-resistant ENC isolates. We hypothesized that EC, KP, and ENC would be representative of other Enterobacterales organisms. PS presents a common nonfermenter with resistance usually caused by a mixture of reduced permeability, ampC beta-lactamase hyperproduction, and efflux. The model will need to be confirmed in a prospective study or at least using a different population. However, given that the majority of patients do not have any of the identified risk factors, many can be managed with first-line antimicrobial agents.

Ultimately, our results have implications for limiting carbapenem use. There is a small population of patients admitted to the intensive care unit without identified risk factors for resistance, but with a high risk of mortality within the first 24–48 hours. This group of patients may need expanded coverage initially until susceptibility to first-line agents can be confirmed. Most of our cohort did not have a history of infection with a resistant organism, but a history of infection within the previous 6 months was identified as a risk factor for primary resistance. Admission from a chronic care hospital or SNF was associated with similar risk. For patients without a high risk of early mortality (eg, absence of septic shock), we suggest that empiric therapy with first-line antimicrobial agents be strongly considered. Organisms can be identified in many cases shortly after growth in culture, although susceptibility testing can take an additional 24–48 hours. At this point, the patient has received empiric antimicrobial therapy for about a day, and there can be clinical decisions regarding the response to treatment and susceptibility. Our study can also inform the design and implementation of clinical decision support tools to limit the overuse of broad antimicrobials, such as carbapenems.

## Conclusion

Patients with Gram-negative bacteremia (EC, KP, ENC, or PS) who are admitted from a chronic care hospital, SNF, or have a history of infection within the prior 6 months, require mechanical ventilation, present with hemiplegia, or have a connective tissue disease are at increased risk of primary cephalosporin resistance. Patients without these risk factors should be strongly considered for ceftriaxone or cefepime treatment rather than treatment with carbapenems or newer broad-spectrum agents.
